# Evaluating Basophil Histamine Content as a Biomarker for Omalizumab Responsiveness in Chronic Spontaneous Urticaria

**DOI:** 10.1111/cea.70327

**Published:** 2026-04-26

**Authors:** Ditte Georgina Zhang, Katrine Baumann, Clive Grattan, Per Stahl Skov, Simon Francis Thomsen

**Affiliations:** ^1^ Department of Dermato‐Venereology & Wound Healing Centre Bispebjerg Hospital Copenhagen Denmark; ^2^ RefLab Hvidovre Denmark; ^3^ St John's Institute of Dermatology, Guy's Hospital London UK; ^4^ Department of Biomedical Sciences University of Copenhagen Copenhagen Denmark

## Abstract

- Our study confirms an association between basophil histamine content and features of type IIb CSU.
- However, this biomarker is not suitable for predicting omalizumab treatment response or treatment course.

To the Editor,

Patients with chronic spontaneous urticaria (CSU) exhibit varying treatment needs to achieve disease control with omalizumab, and adjustments to the standard dose of 300 mg every 4 weeks are common [1]. This calls for biomarkers that may aid physicians in tailoring a more personalised treatment approach [2]. Currently, no reliable biomarkers are available for this purpose. The basophil histamine release assay (BHRA) remains the single best biomarker for identifying (autoimmune) type IIb CSU, which tends to respond slower and less effectively to omalizumab treatment compared to (autoallergic) type I CSU [3, 4]. A previous study reported that low intracellular histamine content in basophils is associated with features of type IIb CSU [5]. This study therefore aimed to explore whether intracellular histamine content of basophils could serve as a stronger or complementary novel biomarker for predicting and monitoring treatment response, offering a simpler and less costly alternative to the BHRA.

A total of 164 adults with CSU who had total cellular blood histamine levels measured were included from the Chronic Urticaria COPenhagen cohort (CU‐COP). Details on cohort recruitment and methods for assessing treatment response have been described previously [1]. Informed consent was obtained from all patients and approval was granted by local authorities (Ref. P‐2020‐318). According to Danish legislation, ethical committee approval was not required. Total cellular blood histamine levels were used to calculate the intracellular histamine content per basophil, as described in detail elsewhere [3]. Continuous variables are presented as medians with interquartile ranges (IQR), and categorical variables as proportions. Correlations between numerical variables were assessed using Spearman's rank correlation coefficient. Group differences were evaluated using the Wilcoxon rank‐sum test, with a *p*‐value < 0.05 considered statistically significant. All analyses were performed using *R* version 4.4.1 and R Studio.

Clinical characteristics of the study cohort are presented in the repository material. The previously reported association between intracellular histamine content per basophil and features of type IIb CSU was replicated. Among the entire cohort, 106 patients initiated omalizumab treatment, of whom 101 were omalizumab‐naïve at baseline. Among these intracellular histamine content per basophil did not differ significantly across patients with fast (within 1 month), delayed (within 3 months) and late/non‐response (after 3 months or no response during treatment) to treatment (Figure 1A). Likewise, the intracellular histamine content per basophil did not differ significantly across the overall treatment response groups when comparing patients with good (symptom‐free or minor symptoms), partial (insufficient response) and poor response (no response) (Figure 1B). When data were stratified according to basophil BHRA status, similar findings were observed, although interpretation is limited by the small numbers of BHRA‐positive patients. Furthermore, intracellular histamine content per basophil did not differ significantly between patients who required dose escalation (defined as an increased dose and/or shortened treatment intervals, *n* = 29) and those who underwent dose reduction (defined as a decreased dose and/or extended treatment intervals, *n* = 38), from the standard dosing regimen, with median values of 1.21 pg/cell and 1.25 pg/cell, respectively (*p* = 0.422). At data lock, 32 patients had discontinued omalizumab treatment, with a median treatment duration of 7 months (IQR: 8). No significant correlation was observed between treatment duration and intracellular histamine content per basophil (*p* = 0.033, *p* = 0.858).

**FIGURE 1:**
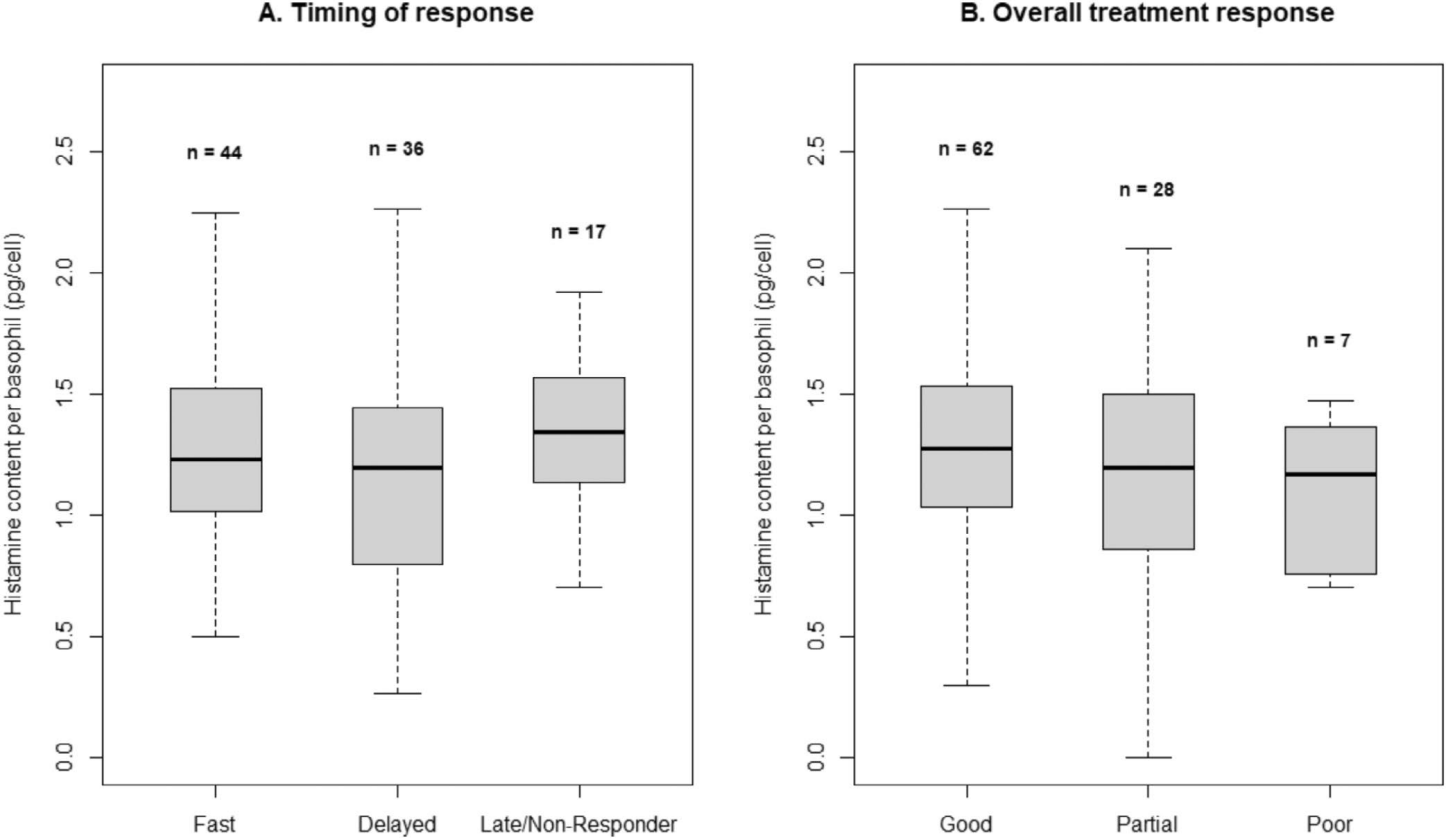
Boxplots of median and interquartile range of intracellular histamine content per basophil stratified by (A) time to treatment response—fast (< 1 month), delayed (< 3 months) and late/non‐responders (≥ 3 months or no response during treatment); and (B) overall treatment response—good (symptom‐free or minor symptoms), partial (insufficient response) and poor (no or lack of response).

While our study confirms that intracellular histamine content per basophil is associated with features of type IIb CSU, it also demonstrates that this biomarker is not suitable for predicting omalizumab treatment response, long‐term dosing requirements or drug survival. Some subgroup analyses in this study involved relatively small sample sizes, which may limit the statistical power and precision of estimates. Additional studies are therefore needed to clarify the relationship between intracellular histamine content of basophils with type IIb features and its potential clinical implications.

## Author Contributions

**Ditte Georgina Zhang:** methodology, formal analysis, investigation, writing – original draft. **Katrine Baumann:** conceptualization, methodology, investigation, writing – review and editing. **Clive Grattan:** writing – review and editing, supervision. **Per Stahl Skov:** conceptualization, methodology, writing – review and editing, supervision. **Simon Francis Thomsen:** conceptualization, writing – review and editing, supervision.

## Funding

The authors have nothing to report.

## Conflicts of Interest

P.S.S. is the Founder and Scientific Advisor of RefLab ApS. S.F.T. has been a speaker and/or served on advisory boards and/or has received research support from Abbvie, Almirall, Boehringer, CSL, Dr. August Wolff, Eli Lilly, Galderma, Incyte, Janssen, Johnson & Johnson, LEO Pharma, Novartis, Novo Nordisk, Pfizer, Sanofi, Servier, Symphogen, UCB and Union Therapeutics. D.G.Z. has nothing to declare. K.B. is employed at RefLab ApS. C.G. has provided consultancy services for Celltrion and Blueprint Medicines, served on an advisory board for ThermoFisher and acted as a meeting delegate for Novartis.

## Data Availability

Additional analyses and results are available in a Zenodo data repository (https://doi.org/10.5281/zenodo.19002174). The data that support the findings of this study are available on request from the corresponding author. The data are not publicly available due to privacy or ethical restrictions.
